# 髓过氧化物酶基因G-463A多态性与肺癌易感性关系的*meta*分析

**DOI:** 10.3779/j.issn.1009-3419.2010.02.08

**Published:** 2010-02-20

**Authors:** 峰 滑, 竞 王, 俊东 谷, 书军 李, 红雨 刘, 清华 周

**Affiliations:** 300052 天津, 天津医科大学总医院, 天津市肺癌研究所, 天津市肺癌转移与肿瘤微环境重点实验室 Tianjin Key Laboratory of Lung CancerMetastasis and Tumor Microenviroment, Tianjin Lung Cancer Institute, Tianjin Medical University General Hospital, Tianjin 300052, China

**Keywords:** 肺肿瘤, 髓过氧化物酶, 多态性, *meta*分析, Lung Cancer, Myeloperoxidase, Gene polymorphism, *meta* analysis

## Abstract

**背景与目的:**

关于髓过氧化物酶基因G-463A多态性与肺癌易感性关系已有广泛研究, 但研究结果不一致。本研究利用*meta*分析的方法探讨髓过氧化物酶基因G-463A多态性与肺癌易感性的相关性。

**方法:**

全面检索公开发表的关于髓过氧化物酶基因G-463A多态性与肺癌易感性关系的研究, 把研究人群分为高加索人群和东亚人群, 以病例组与对照组比值比(odds ratio, OR)为效应指标, 应用*meta*分析软件RevMan 4.2分别计算两种人群的合并OR值及95%CI, 同时给出*meta*分析森林图和倒漏斗图。

**结果:**

纳入文献研究20篇, 其中高加索人群研究纳入病例5 381例, 对照5 827例; 东亚人群研究纳入病例1 558例, 对照1 755例。计算高加索人群及东亚人群髓过氧化物酶基因-463位点AA+AG/GG合并OR值, 分别为0.91(95%CI:0.81-1.02)、0.83(95%CI:0.63-1.09), 高加索人群有发表偏倚, 东亚人群没有发表偏倚。

**结论:**

髓过氧化物酶基因G-463A多态性与肺癌易感性在高加索人群及东亚人群均不具有明显相关性, 东亚人群研究样本量少, 有待进一步研究。

## Abstract (en)

**Background and objective:**

The relationship between myeloperoxidase G-463A genetic polymorphisms and lung cancer susceptibility has been studied extensively.However, the outcomes are not consistent.The aim of this study is to evaluate the relationship between myeloperoxidase genetic polymorphisms and lung cancer susceptibility by *meta* analysis.

**Methods:**

Documents published were retrieved through databases associated with the study.Taking into account the possibilities of heterogeneity of the studies, a statistical test for heterngeneity was performed.The odds ratio and 95%CI were used to evaluate the risks.The *meta* analysis was applied with RevMan software 4.2, and the forest plot and funnel plot of *meta* analysis were worked out.

**Results:**

A total of 5 381 cases and 5 827 controls from studies for Caucasian and a total of 1 558 cases and 1 755 controls from studies for East Asians were included.For Caucasian the pooled OR was 0.91 (95%CI:0.81-1.02);For East Asians, the pooled OR is 0.83 (95%CI:0.63-1.09).Publication bias exits in the study for Caucasian, but not for East Asians.

**Conclusion:**

The results of this study indicated that the polymorphism of myeloperoxidase G-463A was not significantly associated with the lung cancer risk for Caucasian or East Asians.However, further studies for the East Asians is needed for the few subjects.

肺癌是目前在全球范围内对人类健康威胁最大的恶性肿瘤之一, 关于肺癌的基础及临床研究已经成为肿瘤研究领域的重要内容^[1, 2]^。对肺癌病因的研究已经明确, 肿瘤易感性的不同是影响人群患病率的重要因素, 因而对肺癌易感性的研究是当前肺癌研究领域的一个热点, 主要研究方向是单核苷酸多态性与肺癌易感性关系的研究^[3]^。人髓过氧化物酶(myeloperoxidase, MPO)是一种Ⅰ相代谢酶, 广泛存在于肺巨噬细胞内, 具有代谢烟草中的多环芳烃的作用, 因此被认为与肺癌的易感性有关^[4, 5]^。London^[6]^于1997年报告了关于*MPO* G-463A位点与肺癌易感性关系的研究后, 近十余年先后有近30余位研究者报告了这一位点与肺癌易感性的关系的研究, 但结果存有争议, 而且研究人群、样本量、研究方法不一致。本研究采用*meta*分析的方法对已发表的研究结果进行系统评价, 以进一步评价*MPO*-463位点多态性与肺癌易感性的关系。

## 1 资料与方法

### 1.1 文献检索

检索中国学术期刊网全文数据库(CNKI)、万方数据库、维普数据库、Pubmed、Ovid、Elsevier数据库, 收集国内外公开发表的“关于*MPO*-463位点多态性与肺癌易感性的关系”的独立病例对照研究, 中文文献检索词为:“髓过氧化物酶”、“MPO”、“myeloperoxidase”、“肺癌”、“肺肿瘤”, 分别作为关键词、自由词、主题词进行检索; 英文文献检索词为:“MPO” “myeloperoxidase”、“lung cancer”、“lung neoplasm”, 分别作为主题词、自由词进行检索。为尽量避免漏查文献, 对所有检索文献中提供的参考文献进行二次检索, 相关综述文章、有关会议的摘要均被检索, 以发现可能的合格研究。以上检索工作由3个研究者独立完成, 文献检索截止日期为2009年11月18日。

### 1.2 文献纳入标准

① 研究方法符合流行病学要求; ②基因分型方法可信; ③文献提供基因型频数、比值比(odds ratio, OR)及95%CI; ④研究人群为高加索人群及东亚人群。

### 1.3 文献剔除标准

① 研究方法不符合流行病学要求; ②基因分型技术不可信, 研究质量较差; ③重复研究人群资料; ④不能提供原始数据资料; ⑤其他研究人群。

### 1.4 数据提取

3个研究者独立提取纳入的每个研究的相关数据, 如有异议, 与第4个研究者协商解决, 然后汇总数据, 提取以下内容:①文献作者、研究对象国家和种族背景、发表年代和杂志名称; ②纳入研究总的例数, 病例和对照组研究对象的定义和特点; ③病例和对照组的等位基因和基因型的分布情况; ④基因分析样本来源和采用的方法; ⑤病例和对照组研究对象是否符合*Hardy-Weinberg*遗传平衡。

### 1.5 统计学分析

按照*meta*分析的要求整理原始文献并摘录数据, 对各研究的基因分布进行*Hardy-Weinberg*遗传平衡检验。由于部分研究仅提供了GG、AG+AA两种基因型分布的频数, 而且AA型在人群中的比例很低, 本研究只进行了AA+AG/GG两种基因型比较的系统评价。应用统计分析软件Review manager 4.2分析AA+AG/GG的OR值及95%可信区间(95% confidence interval, 95%CI), 绘制OR值分布图。对所得的OR值取自然对数In(OR), 进行齐性检验, 根据一致性结果选择相应的数据合并方法。若各研究结果间无异质性, 则采用*Mantel-Haenszel*固定效应模型进行数据合并, 计算合并OR及95%CI; 若结果间存在异质性, 则选用*Dersimonian-Laird*随机效应模型计算。进行敏感性分析, 绘制漏斗图并采用*Egger*法对发表结果是否偏倚进行评估。

## 2 结果

### 2.1 纳入分析研究的一般资料

根据文献纳入标准, 共纳入文献20篇, 其中来自欧洲高加索人群报道13篇, 中国人群报道3篇, 韩国人群3篇, 日本及高加索人群混合研究1篇, 纳入文献的发表时间为1997年-2008年, 病例诊断均符合WHO标准, 基因分型均使用公认的、科学的检测方法。14项研究进行了性别配对, 16项进行了年龄配对。一般资料见表 1。

**1:** 纳入分析的20项研究一般资料 The characteristic of the studies included

First anthor (reference NO)	Year	No. of cases	No. of controls	Mean age of cases	Male %, case	Tobacco %, case	Histology			Method	Maching critirian	Ethic
							sc	ac	other			
Wu^[7]^	2003	98	112	60.8±9.8	76.5	59.2	unknown			RFLP	Age, gender	Chinese
Lv^[8]^	2002	314	320	58.6±9.7	69.8	54.9	56.4	43.6	0	sscp	Age, gender	Chinese
Yang^[9]^	2007	318	353	55.4±9.6	67.6	65.1	22.3	54.7	23	RFLP	none	Korean
Park^[10]^	2006	432	432	61.6±9.0	81.5	82.4	48.6	32.6	18.8	RFLP	Age, gender	Korean
Chan^[11]^	2005	75	162	unknown	unknown	unknown	unknown			RFLP	Age, gender	Chinese
London^[6]^	1997	182	459	63.6	unknown	95.6	unknown			RFLP	Age	Caucasian
Marchand^[12]^	2000	108	163	unknown	unknown	unknown	unknown			RFLP	Age, gender	Japanese
Marchand^[12]^	2000	135	171	unknown	unknown	unknown	unknown			RFLP	Age, gender	Caucasian
Cascorbi^[13]^	2000	196	196	63	76.5	unknown	unknown			RFLP	Age, gender	Caucasian
Feyler^[14]^	2002	150	172	58.4	93	100	65	35	0	RFLP	Gender	Caucasian
Xu^[15]^	2002	988	1 128	unknown	54	94	24	50	26	RFLP	none	Caucasian
Dally^[16]^	2002	625	340	60.0	77.3	unknown	36	36	28	probes	Age, gender	Caucasian
Kantarci^[17]^	2002	307	307	67	58.3	85.2	unknown			RFLP	none	Caucasian
Larsen^[18]^	2006	627	624	63.4±9.4	71.9	94	45.1	45.2	9.7	RFLP	Age, gender	Caucasian
Schabath^[19]^	2002	375	378	62.1	52	83.2	14.4	39.2	46.4	RFLP	Age, gender	Caucasian
Salazar^[20]^	2003	110	119	59.9±9.8	57	100	52	41	7	RFLP	Age, gender	Caucasian
Liu^[21]^	2004	830	1 119	65	unknown	unknown	unknown			RFLP	Age, gender	Caucasian
Chevrier^[22]^	2003	243	245	59.4±9.6	100	100	46	24	30	probes	Age, gender, rseidence	Caucasian
Misra^[23]^	2001	315	311	60	100	100	unknown			probes	Age	Caucasian
Zienolddiny^[24]^	2008	297	258	65	unknown	unknown	unknown			sequencing	Age, gender, smoking	Caucasian
Yoon^[25]^	2008	213	213	57	0	0	3.3	81.2	15.5	probes	Age	Korean

### 2.2 高加索人群*MPO* G-463A与肺癌关联性分析

#### 2.2.1 *meta*分析结果

纳入数据的欧洲高加索人群13项研究累计病例5 381例, 对照5 827例。通过对各研究的AA+AG/GG结果的异质性检验, 得*χ*^2^值为25.17(*P*=0.02), 认为有统计学意义, 故采用随机效应模型进行分析得合并OR值为0.91(95%CI:0.81-1.02), *z*值为1.68(图 1)。

**1:**
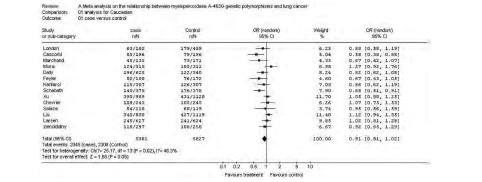
高加索人群MPO G-463A与肺癌关联性分析森林图 The forest plot of the *meta* analysis for the Caucasians

#### 2.2.2 敏感性分析

换用固定效应模型, 改用统计效应指标为Peto odds ratio, 合并OR值未见明显改变, 剔除样本量最大的Xu等^[15]^的研究后, 计算合并OR值未见明显改变, 提示结果稳定。

#### 2.2.3 发表偏倚的评估

发表偏倚评估所得漏斗图的图形对称性差, 应用SAS程序拟合*Egger*检验, 提示有发表偏倚(*t*=2.67, *P*=0.02)(图 2)。

**2:**
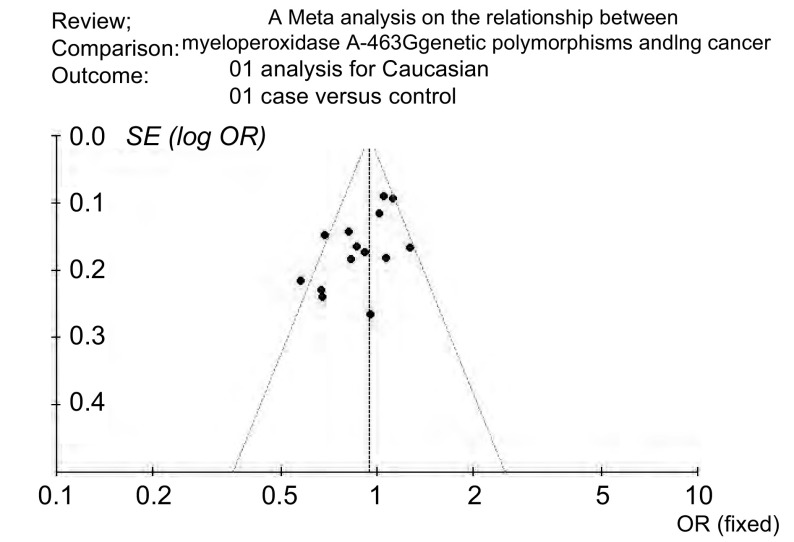
高加索人群MPO G-463A与肺癌关联性分析漏斗图 The funnel plot of the *meta* analysis for the Caucasians

### 2.3 东亚人群*MPO* G-463A与肺癌关联性分析

#### 2.3.1 *meta*分析结果

纳入数据的亚洲人群7项研究累计病例1 558例, 对照1 755例。通过对各研究的AA+AG/GG结果的异质性检验, 得值为14.83(*P*=0.02), 认为有统计学意义, 故采用随机效应模型进行分析得合并OR值为0.83(95%CI:0.63-1.09), *z*值为1.35(图 3)。

**3:**
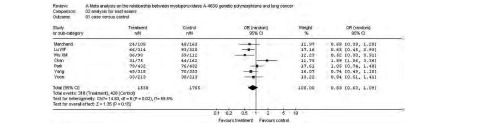
东亚人群MPO G-463A与肺癌关联性分析森林图 The forest plot of the *meta* analysis for East Asians

#### 2.3.2 敏感性分析

改用固定效应模型计算, 合并OR值为0.82, 未见明显改变, 改用统计效应指标为Peto odds ratio, 合并OR值未见明显改变, 剔除样本量最大的Park等^[10]^的研究后, 计算合并OR值未见明显改变, 提示结果稳定。

#### 2.3.3 发表偏倚的评估

发表偏倚评估所得漏斗图的图形基本对称, 应用SAS程序拟合*Egger*检验, 未见明显发表偏倚(*t*=-0.39, *P*=0.71)(图 4)。

**4:**
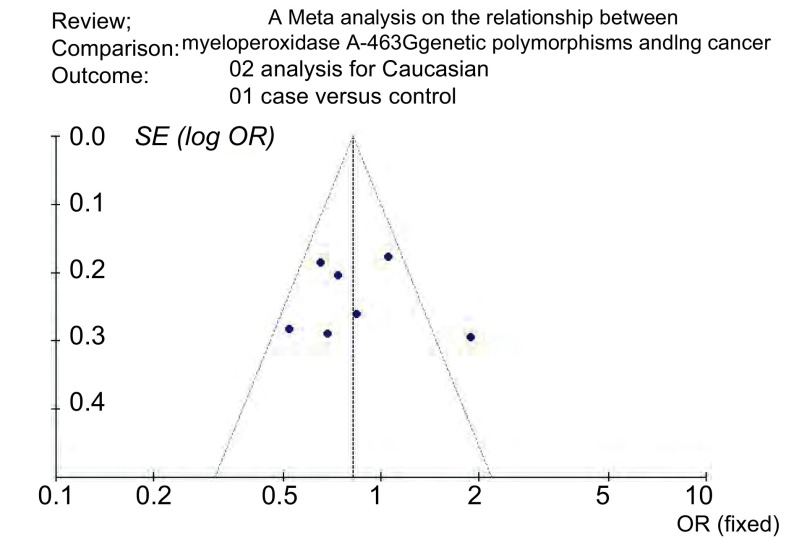
东亚人群MPO G-463A与肺癌关联性分析漏斗图 The funnel plot of the *meta* analysis for the East Asians

## 3 讨论

考虑到MPOG-463A在不同种族的差异分布(http://www.ncbi.nlm.nih.gov/projects/SNP/snp ref.cgi?rs=2333227), 本研究只收集以欧洲高加索人群及东亚中日韩人群为研究对象的文献进行系统评价, 检索到符合要求的文献28篇。其中重复报道文献3篇, 2篇没有提供详细基因频数, 2篇为系统评价, 1篇研究质量较差, 予以剔除。共计纳入研究文献20篇, 其中高加索人群研究13篇, 东亚人群研究6篇, 1篇研究人群为高加索及日本人群^[12]^。分析各个独立研究样本人群的流行病学资料, 可以看到纳入研究人群的性别、年龄、地域、吸烟状况、饮食习惯、职业暴露、肺癌病理类型等具有明显差异性, 其中11项研究^[6, 7, 11-14, 20, 22, 24, 25]^的病例样本量小于300, 距离遗传关联性研究对样本量的要求有一定差距, 因此单个独立研究所得到的结果有可能会是假阳性或者假阴性。对两种人群发表偏倚的评价, 从所作出的漏斗图形态及参考*Egger*法所计算出的t值, 认为在高加索人群研究中有发表偏倚, 但不明显, 在东亚人群研究中没有偏倚。由于部分文献只给出了AG+AA基因型的频数, 而且AA为少见基因型, 本研究以AA+AG/GG的合并OR值为效应指标。

对高加索人群的分析, 采用固定效应模型计算得到的合并OR值为0.94(95%CI:0.87-1.02), 异质性检验P值为0.02, 改用随机效应模型, 计算合并OR值为0.91(95%CI:0.81-1.02), 由于部分进行亚组人群分析的研究分类标准不一致, 无法进行亚组人群的*meta*分析。剔除样本量最大的Xu等^[15]^的研究, 结果无明显改变, 提示敏感性分析得到的结果是稳定的。对东亚人群的分析, 采用固定效应模型计算得到的合并OR值为0.82(95%CI:0.70-0.97), 异质性检验*P*值为0.02, 改用随机效应模型, 计算合并OR值为0.83(95%CI:0.63-1.09)。有3篇文献进行了亚组分析, 但分组不同, 而且样本量有限, 所以未进行亚组人群的*meta*分析。剔除样本量最大的Park^[10]^的研究, 结果无明显改变, 提示敏感性分析的到的结果是稳定的。

Taioli等^[14, 26, 27]^对这一问题先后做了系统评价。其中Feyler于2002年第一次做了*meta*分析, 对高加索人群分析计算的结果是0.82(95%CI:0.66-1.02), 共纳入研究6篇。Kiyohara仅仅计算了AA基因型的合并OR值为0.70。Taioli进一步研究了此问题, 对纳入的15篇文献进行*meta*分析计算的合并OR值为0.91(95%CI:0.84-0.99), 研究人群主要为高加索人群。他利用其中的10篇文献^[6, 12-14, 16, 18-20, 22, 28]^进行的pooled analysis得出的AG基因型OR值为0.88(95%CI:0.80-0.97), AA基因型的合并OR值为0.71(95%CI:0.57-0.88), 但人群为混合人群, 而且其中有2篇文献^[13, 20]^采用的数据与公开发表文献上的数据有差异。总的来说, 这些研究的结果与我们是一致的。

髓过氧化物酶是一种糖蛋白, 主要存在于白细胞中, 大量的体外细胞实验、动物实验以及以人体为对象的实验研究提示髓过氧化物酶的异质性可能与肺癌易感性具有潜在的关联^[29-31]^。通过对已发表文献的系统评价, 我们认为髓过氧化物酶基因G-463A位点两种基因型在肺癌人群中分布具有差异性, 但在总体人群中的潜在作用是微弱的, 对单个基因多态性的研究, 意义是十分有限的。
